# Impact of COVID-19 vaccination on the risk of developing long-COVID and on existing long-COVID symptoms: A systematic review

**DOI:** 10.1016/j.eclinm.2022.101624

**Published:** 2022-08-27

**Authors:** Kin Israel Notarte, Jesus Alfonso Catahay, Jacqueline Veronica Velasco, Adriel Pastrana, Abbygail Therese Ver, Flos Carmeli Pangilinan, Princess Juneire Peligro, Michael Casimiro, Jonathan Jaime Guerrero, Ma. Margarita Leticia Gellaco, Giuseppe Lippi, Brandon Michael Henry, César Fernández-de-las-Peñas

**Affiliations:** a Department of Pathology, Johns Hopkins University School of Medicine, Baltimore, MD, USA; b Department of Medicine, Saint Peter's University Hospital, New Brunswick, NJ, USA; c Faculty of Medicine and Surgery, University of Santo Tomas, Manila, Philippines; d Learning Unit 3, College of Medicine, University of the Philippines Manila, Manila, Philippines; e Section of Clinical Biochemistry, University of Verona, Verona, Italy; f Clinical Laboratory, Division of Nephrology and Hypertension, Cincinnati Children's Hospital Medical Centre, OH, USA; g Department of Physical Therapy, Occupational Therapy, Physical Medicine and Rehabilitation, Universidad Rey Juan Carlos, Madrid, Spain

**Keywords:** Post-COVID syndrome, Long-COVID symptoms, Vaccine, SARS-CoV-2

## Abstract

**Background:**

Although COVID-19 vaccination decreases the risk of severe illness, it is unclear whether vaccine administration may impact the prevalence of long-COVID. The aim of this systematic review is to investigate the association between COVID-19 vaccination and long-COVID symptomatology.

**Methods:**

MEDLINE, CINAHL, PubMed, EMBASE, and Web of Science databases, as well as medRxiv and bioRxiv preprint servers were searched up to June 20, 2022. Peer-reviewed studies or preprints monitoring multiple symptoms appearing after acute SARS-CoV-2 infection either before or after COVID-19 vaccination collected by personal, telephone or electronic interviews were included. The methodological quality of the studies was assessed using the Newcastle-Ottawa Scale.

**Findings:**

From 2584 studies identified, 11 peer-reviewed studies and six preprints were included. The methodological quality of 82% (*n*=14/17) studies was high. Six studies (*n*=17,256,654 individuals) investigated the impact of vaccines before acute SARS-CoV-2 infection (vaccine-infection-long-COVID design). Overall, vaccination was associated with reduced risks or odds of long-COVID, with preliminary evidence suggesting that two doses are more effective than one dose. Eleven studies (*n*=36,736 COVID-19 survivors) investigated changes in long-COVID symptoms after vaccination (infection-long-COVID-vaccine design). Seven articles showed an improvement in long-COVID symptoms at least one dose post-vaccination, while four studies reported no change or worsening in long-COVID symptoms after vaccination.

**Interpretation:**

Low level of evidence (grade III, case-controls, cohort studies) suggests that vaccination before SARS-CoV-2 infection could reduce the risk of subsequent long-COVID. The impact of vaccination in people with existing long-COVID symptoms is still controversial, with some data showing changes in symptoms and others did not. These assumptions are limited to those vaccines used in the studies.

**Funding:**

The LONG-COVID-EXP-CM study supported by a grant of Comunidad de Madrid.

**Research in context**

**Evidence before this study**

We searched PubMed and Web of Science databases for studies published until April 1, 2022, using keywords “long-COVID”, OR “post-COVID” AND “vaccine” OR “vaccination”. We identified different studies analyzing the impact of COVID-19 vaccination in long COVID symptoms, but no systematic review was available in the literature.

**Added value of this study**

This first systematic review evaluating evidence to date about the impact of vaccines on long COVID supports that vaccination before SARS-CoV-2 infection is able to reduce the risk of developing long-COVID. The impact of vaccination in people with long-COVID symptomatology is controversial, with data showing changes in symptoms and others did not.

**Implications of all the available evidence**

Current results support that COVID-19 vaccines can be used as preventive strategy for decreasing the risk of long-COVID, but data about its effects on people with current long-COVID needs further research. Questions about the impact on hospitalised/non-hospitalised, males/females and the impact of vaccine boosters is clearly needed.

Alt-text: Unlabelled box

## Introduction

COVID-19 caused by SARS-CoV-2 is the deadliest communicable healthcare outbreak of the 21^st^ century. COVID-19 vaccines have significantly reduced the risk of developing the severe or critical forms of disease, as well as mortality brought by COVID-19.^1^ Nonetheless, vaccines seem unable to fully reduce the spread of SARS-CoV-2 variants of concerns (VOCs).^2^

Following the COVID-19 outbreak, leading to hundreds of millions of acute cases and six million deaths, healthcare professionals are in front of another crisis brought about by development and/or persistence of symptoms after the acute phase of SARS-CoV-2 infection (typically after 3 months), a condition conventionally called long-COVID^3^ or post-COVID.^4^ More than 100 symptoms can appear after a SARS-CoV-2 acute infection, affecting multiple systems, *e.g.,* cardiovascular, respiratory, musculoskeletal, or neurological.^5^ Several meta-analyses observed that almost 50% of COVID-19 survivors had a lingering plethora of symptoms lasting for weeks or months^6^, ^7^, ^8^ but also one year^9^^,^^10^ after SARS-CoV-2 infection.

As of August 2022, more than 12.4 billion COVID-19 vaccine doses have been administered globally.^11^ Although vaccination decreases the risk of severe COVID-19, it is unclear whether vaccination before or after an acute infection improves or reduces the prevalence of long-COVID symptoms. In fact, vaccinated people can still be infected and suffer from asymptomatic, mild or moderate COVID-19, especially when the infection is sustained by VOCs (namely Omicron). Since long-COVID can arise even after a mild or asymptomatic SARS-CoV-2 infection,^12^ it is in question what real impact vaccines will have on long-COVID.^13^, ^14^, ^15^, ^16^ This review is the first to date to systematically investigate the impact of COVID-19 vaccination on long-COVID symptoms. Therefore, the research question of this review was: “what is the impact of COVID-19 vaccines on the risk of developing long-COVID or on existing long-COVID in COVID-19 survivors?

## Methods

This systematic review adheres to the Preferred Reporting Items for Systematic Reviews and Meta-Analyses (PRISMA) statement,^17^ and was prospectively registered in the Open Science Framework (OSF) database (https://osf.io/34djr). No ethical committed is needed for a systematic review.

### Search strategy and selection criteria

Electronic literature searches were conducted by two different authors on the following databases: MEDLINE, CINAHL, PubMed, EMBASE, and Web of Science databases, as well as on preprint servers medRxiv and bioRxiv, for studies published until June 20, 2022. Database search strategies were conducted with assistance of an experienced health science librarian. We also screened the reference list of identified papers for capturing black literature. Searches were limited to human studies and English language citations by using the following combinations of terms: “long-COVID”, “long-COVID symptoms”, “long hauler”, “post-COVID-19” OR “post-acute COVID-19 syndrome” OR “post-acute COVID-19 symptoms” OR “COVID-19 sequelae” AND “vaccine” OR “vaccination” OR “COVID-19 vaccines” OR “SARS-CoV-2 vaccine”. The search strategy combined these terms using Boolean operators for the main databases is detailed in Supplementary Table.

The inclusion and exclusion criteria were formulated using the Population, Intervention, Comparison and Outcome (PICO) principle:

*Population:* Adults (>18 years) infected by SARS-CoV-2 and diagnosed with real-time reverse transcription-polymerase chain reaction (RT-PCR) assay. Individuals could have been hospitalised or not by SARS-CoV-2 acute infection.
*Intervention:* Any type of COVID-19 vaccine. We included the following types of COVID-19 vaccines: BNT162b2 (“Pfizer/BioNTech”), AZD1222 (“Oxford-AstraZeneca”), mRNA-1273 (“Moderna”), and Ad26.COV2.S (“Janssen”). Vaccine doses can be administered before or after SARS-CoV-2 acute infection.
*Comparison*: Individuals not receiving any COVID-19 vaccine.
*Outcome*: Collection of multiple symptoms (post-COVID-19 or long-COVID) developed after a SARS-CoV-2 acute infection (https://www.nhs.uk/conditions/coronavirus-covid-19/long-term-effects-of-coronavirus-long-covid/) by personal, telephone, or electronic interviews. We included any type of symptom appearing after the infection e.g., physical (fatigue, pain), cognitive (brain fog, memory loss), respiratory (dyspnea, palpitations, cough), gastrointestinal (diarrhoea, stomachache, vomiting) or mental problems (depression, anxiety, sleep disturbances). Due to the different definitions of long-COVID, no specific follow-up period for the presence of symptoms after the acute infection was determined. Studies monitoring solely changes in immunologic or serologic biomarkers without assessment of post-COVID symptoms were excluded.

This review included observational cohort, cross-sectional, and case-control studies where samples of COVID-19 survivors, either hospitalised or non-hospitalised, were followed for presence of symptoms appearing after a SARS-CoV-2 acute infection before or after COVID-19 vaccination. Editorials, opinion, and correspondence articles were excluded.

Two authors reviewed the title and abstract of those publications identified in the databases. Duplicates were then removed. The title and abstract were screened for eligibility and posterior full-read text. Data including authors, country, sample size, setting, vaccination status, type of vaccine, clinical data, and post-COVID symptoms before and after vaccination were extracted from each study. Authors had to reach consensus on data extraction. Discrepancies between reviewers at any stage of screening process were resolved by asking a third author, when necessary.

### Data analysis

The methodological quality of the studies was independently assessed by two authors using the Newcastle-Ottawa Scale, a star rating system evaluating the risk of bias of case-control and cohort studies.^18^ The Newcastle-Ottawa Scale evaluates the following sections in cohort studies: case selection (*i.e.,* representativeness of the cohort, selection of non-exposed cohort, case definition, outcome of interest), comparability (*i.e.,* proper comparison by controlling for age, gender, or other factors, between-groups) and exposure (*i.e.,* outcome assessment, long enough follow-up, adequate follow-up). Some of these items are adapted if the studies used case-control design. For instance, case selection item includes adequate case definition or selection of controls. In cohort studies using longitudinal design or case-control studies, a rating of 7 to 9 stars indicates high quality, 5 to 6 medium quality, and less than or equal to 4 is of low quality. In cohort studies using cross-sectional design, a maximum of 3 stars can be awarded. Studies scoring 3 stars are considered of good quality, 2 stars of fair quality, and 1 star of poor quality. Methodological quality was initially evaluated by two authors. If there is disagreement, a third researcher arbitrated a consensus decision.

Meta-analysis was not deemed appropriate due to the high heterogeneity between studies. Accordingly, we conducted a synthesis of the data reported by addressing population, vaccine status related to acute infection, limitations, and methodological quality.

### Role of the funding source

The sponsor had no role in the design, collection, management, analysis, or interpretation of the data, draft, review, or approval of the manuscript or its content. The authors were responsible for the decision to submit the manuscript for publication, and the sponsor did not participate in this decision. All authors had access to the data. Kin Israel Notarte and César Fernández-de-las-Peñas verified the data set. All authors were responsible for making the decision to submit this manuscript.

## Results

### Study selection

The electronic search identified 2584 titles for initial screening. After removing duplicates (*n*= 138) and papers not directly related to vaccines and long-COVID (*n*=2396), 50 studies remained for abstract examination. 29 were excluded after abstract examination: not available in English text (*n*=3), case reports and case series studies (*n*=5), review articles (*n*=7), full text not available (*n*=4), and not focused on vaccines and long-COVID (*n*=10).

**Figure 1:**
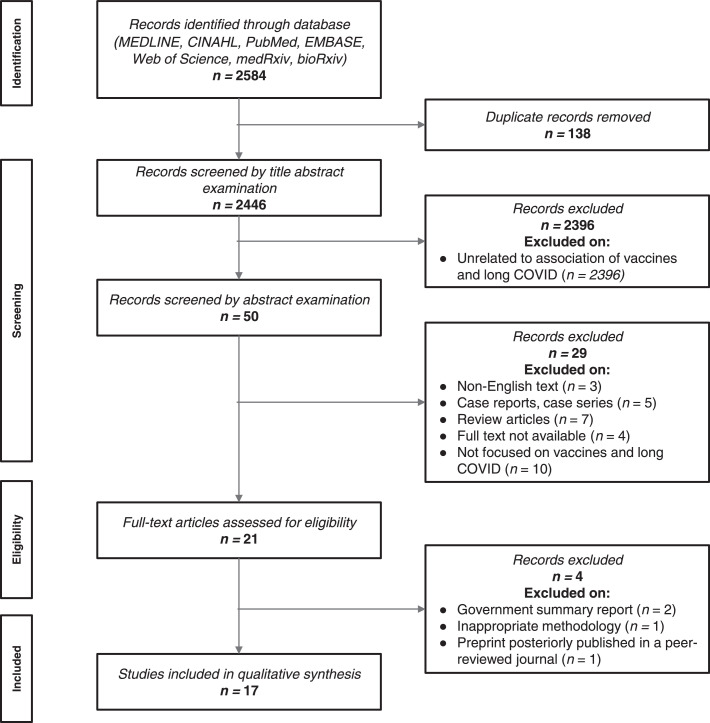
Preferred Reporting Items for Systematic Reviews and Meta-Analyses (PRISMA) Flow diagram.

A total of 13 published and 8 preprint full-text articles were assessed for eligibility^19^, ^20^, ^21^, ^22^, ^23^, ^24^, ^25^, ^26^, ^27^, ^28^, ^29^, ^30^, ^31^, ^32^, ^33^, ^34^, ^35^, ^36^, ^37^, ^38^ (Figure 1). Two articles were excluded because they were government summary reports.^36^^,^^37^ One preprint was excluded because it was a study protocol.^39^ Lastly, one preprint^38^ was excluded because the same study was previously published in a peer-reviewed journal.^23^ Finally, a total of 11 peer-reviewed studies and 6 preprints were included in the systematic review.^19^, ^20^, ^21^, ^22^, ^23^, ^24^, ^25^, ^26^, ^27^, ^28^, ^29^, ^30^, ^31^, ^32^, ^33^, ^34^, ^35^

### Study characteristics

We identified two types of studies according to the relationship between vaccination and acute infection: (1) studies investigating the development of long-COVID symptoms in people who had received COVID-19 vaccine before being infected (vaccine - infection - long COVID); and (2) studies investigating changes in long-COVID symptoms in people who had previously been infected, developed long-COVID, and then received vaccine after (infection - long COVID - vaccine).

The characteristics of the ‘vaccine - infection - long COVID’ studies are shown in Table 1 (total sample *n*=17,256,654 participants). Five^19^^,^^20^^,^^22^, ^23^, ^24^ out of six articles provided data on mRNA and vector vaccines while the remaining study^21^ did not list the specific vaccine included. The countries of origin for these studies were the United States of America (USA), United Kingdom (UK), and India. Three papers^20^, ^21^, ^22^ investigated patients who have had at least 2 doses of vaccine while the remaining three^19^^,^^23^^,^^24^ papers only required at least one dose of vaccine.

**Table 1:** Summary of results for ‘vaccine - infection - long COVID’ studies.

Author and Country of Origin	Study Design and Study Period	Sample Size	Median Age (Range)	Cases	Controls	Objective Assessment of Symptoms	Post-Acute Symptoms Reported	Vaccine Information (Product, Dose, Follow-up Period)	Impact of Vaccine on Symptoms Associated with long-COVID
Simon et al. 2021^19^ United States of America	Retrospective cohort Study Period: ND	*n* = 2392 Female = 1504 Hospitalized = 175	0 to >65 year Median age: ND	2392 vaccinated	2392 unvaccinated	Arcadia Data Research	Chest Pain Palpitations Altered mental state Anorexia Chills Fatigue Fever Malaise Loss of sense of Smell Loss of sense of taste Nasal congestion Sore throat Abdominal pain Diarrhoea Digestive changes Nausea and/or Vomiting Arthralgia Muscle weakness General weakness Myalgia Headache Cough Dyspnea	Product: BNT162b2, mRNA 1273, Ad26.COV2.S Dose: at least one Follow-up: 20 weeks	OR (95%CI) Any symptom Prior to COVID-19 OR 0.22 (0.196–0.245) >1 symptom Prior to COVID-19 OR 0.113 (0.09–0.143)
Antonelli et al. 2022^20^ United Kingdom	Case control December 8, 2020 to July 4, 2021	*n* = 9462	Mean age: 52.9 years	Individuals with positive COVID-19 test at least 14 days after their first vaccination dose or 7 days after their second vaccination dose and had no positive test before vaccination	Unvaccinated participants reporting a positive SARS-CoV-2 test	COVID-19 Symptom Study App (UK Department of Health and Social Care)	Fever Persistent cough Loss of smell Fatigue Headache Sore throat Dizziness Chills or shivers Hoarse voice Brain fog Unusual muscle pains Eye soreness Diarrhoea Shortness of breath Chest pain Nausea Tinnitus Abdominal pain Earache	Product: BNT162b2, ChAdOx1 nCoV-19, and mRNA 1273 Dose: Two doses Follow-up: At least 14 days after first dose of vaccination and at least 7 days after second dose of vaccination	OR (*p*-value) All age groups Symptoms lasting ≥28 day D1: 1.03 (0.78) D2: 0.51(0.006) Younger adults (18–59 years) Symptoms lasting ≥28 day D1: 1.22 (0.14) D2: 0.37 (0.025) Older adults (60+ years) Symptoms lasting ≥28 day D1: 0.87 (0.29) D2: 0.56 (0.044)
Senjam et al. 2021^21^ India	Cross-sectional June 16 to July 28, 2022	*n* = 773 Female = 337 Male = 436 Hospitalized = 51	Median age: 34 years	366 vaccinated	407 unvaccinated	A semi-structured questionnaire was developed for the study purpose. The questionnaire was digitized using Google forms.	Fatigue Joint pain Muscle Hair loss Headache Breathlessness Sleep disturbance Cough	Product: Not reported Dose: Two doses Follow-up: Not reported	aOR (95%CI) Vaccinated: OR 0.65 (0.45–0.96) Unvaccinated: OR 0.55 (0.37–085)
Ayoubkhani et al. 2022^22^ United Kingdom	Prospective Cohort Study Period: ND	*n* = 6180 Female = 3335 Hospitalized = N/A	Mean (SD) Vaccinated: 49.0 (12.0) years Unvaccinated: 46.7 (11.2) years	3090 double vaccinated	3090 unvaccinated	UK COVID-19 Infection Survey	ND	Product: ChAdOx1 nCoV-19, BNT162b2, and mRNA 1273 Dose: Two doses Median follow-up Vaccinated: 96 days (IQR: 90 to 104) Unvaccinated: 98 days (IQR: 89 to 109)	aOR (95%CI) Long-COVID of any severity: aOR 0.59 (0.50 to 0.69)
Al-Aly et al. 2022^23^ United States of America	Retrospective cohort March 1, 2020 and January 15, 2021	*n* = 13,369,073 BTI: *n*=33,940 Contemporary controls n = 4,983,491 Historical controls *n* = 5,785,273 Vaccinated controls n = 2,566, 369 Females = 1,300, 744 Hospitalized = 4478	BTI: 66.6 (13.8) years SARS-COV-2 infection: 57.8 (15.9) years Contemporary control: 63.3 (16.6) years Vaccinated control: 67.7 (14.3) years Historical control: 61.8 (17.3) years	33,940 vaccinated with BTI BNT162b2*n*=16,271 mRNA 1273 *n*=13,726 Ad26.COV2.S *n*=3943	People with SARS-CoV-2 infection and no prior history of vaccination *n* = 1,13,474	National healthcare databases of the US Department of Veterans Affairs	Cardiovascular, coagulation and hematologic gastrointestinal kidney mental health metabolic musculoskeletal neurologic disorders	Product: Ad26.COV2.S Dose: One Product: BNT162b2 Dose: Two Product: mRNA 1273 Dose: One Follow-up: within 6 months	BTI: Risk of death HR: 0.66 (0.58–0.74) burden of -10.99 (−13.45 to −8.22) Post-acute sequelae HR = 0.85 (0.82, 0.89) burden of -43.38 (−53.22 to −33.31) **negative values denote reduced burden in BTI relative to SARS-CoV-2 infection
Taquet et al. 2022^24^ United States of America	Retrospective Cohort January 1, 2021 to August 31, 2021	*n* = 18,958 Female = 11,437 Hospitalized = No Data	Mean (SD), at infection: Vaccinated: 56.5 (18.0) years Unvaccinated: 57.6 (20.6) years	9479 participants vaccinated with COVID-19 vaccine	9479 participants unvaccinated with COVID-19 vaccine but with influenza vaccine at any time	TriNetX Analytics (Federated Network of Linked Electronic Health Records)	Abdominal symptoms Abnormal breathing Anxiety/Depression Chest/Throat Pain Cognitive symptoms Fatigue Headache Myalgia Other pain	Product: BNT162b2, mRNA 1273 Ad26.COV2.S, unspecified subtype Dose: 1-2 Follow-up: within 6 months	Fatigue (HR 0.89, 95% CI 0.81–0.97) Myalgia (HR 0.78, 95% CI 0.67-0.91) Pain (HR 0.90, 95% CI 0.81-0.99) Abnormal breathing (HR 0.89, 95% CI 0.81–0.98) Cognitive symptoms (HR 0.87, 95% CI 0.76–0.99) HR for other symptoms were not reported

ND - no data; aOR - adjusted odds ratio; SD - standard deviation; OR - odds ratio; HR - hazard ratio; RR - risk ratio; BTI - breakthrough infections

For the ‘vaccine - infection - long COVID’ studies, the impact of vaccine on long-COVID symptoms was presented as odds ratio (OR), adjusted odds ratio (aOR), and hazards ratio (HR). Two articles^23^^,^^24^ used HR, two 19^20^ used purely OR, one^22^ used aOR, and another^21^ used both aOR and OR for expressing differences in long-COVID development between vaccinated and non-vaccinate people.

Overall, all six articles^19^, ^20^, ^21^, ^22^, ^23^, ^24^ agreed that vaccination before SARS-CoV-2 acute infection was associated with reduced risks or odds of long-COVID. There was high heterogeneity in the time from vaccination to infection, suggesting that people who had been vaccinated a month before being infected has lower risk of developing long-COVID symptoms. Antonelli et al.^24^ and Taquet et al.^24^ further posit that two doses could be more effective for reducing the risk of long-COVID than a single dose. Al-Aly et al.^24^ concluded that BNT162b2 (“Pfizer/BioNTech”) and mRNA-1273 (“Moderna”) vaccines were more effective for mitigating the risk of long-COVID compared to Ad26.COV2.S (“Janssen”) vaccine. Five^19^, ^20^, ^21^^,^^23^^,^^24^ papers listed specific symptoms, while the remaining^22^ did not specify any particular post-COVID symptom. The most common post-COVID symptoms analysed in the ‘vaccine-infection-long COVID’ papers were fatigue (*n*=5), muscle and joint pain (*n*=5), abdominal pain (*n*=4), diarrhoea (*n*=4), along with cough (*n*=4). Neurological symptoms and mental health problems including headache (*n*=4), brain fog or memory loss (*n*=2), anxiety (*n*=2), depression (*n*=1), altered mental state (*n*=2), and mood disorder (*n*=1) were also noted.

The characteristics of the ‘infection - long COVID - vaccine’ studies are shown in Table 2, involving 36,736 COVID-19 survivors and encompassing eleven papers.^25^, ^26^, ^27^, ^28^, ^29^, ^30^, ^31^, ^32^, ^33^, ^34^, ^35^ With respect to the geographical distribution, four articles were from the UK, two from the USA, one each from France, Italy, Israel, Japan, and Switzerland. Three out of 11 articles^26^^,^^32^^,^^33^ gathered data on mRNA vaccines only, seven articles^25^^,^^27^^,^^29^, ^30^, ^31^^,^^34^^,^^35^ on mRNA and viral vector vaccines, while one article^28^ did not mention the type of vaccine. All studies included patients with at least a single dose of vaccine.

**Table 2:** Summary of results for ‘infection - long COVID - vaccine’ studies.

Author and Country of Origin	Study Design and Study Period	Sample Size	Median Age (Range)	Cases	Controls	Objective Assessment of Symptoms	Post-Acute Symptoms Reported	Vaccine Information (Product, Dose, Follow-up Period)	Impact of Vaccine on Symptoms Associated with long-COVID
Arnold et al. 2021^25^ United Kingdom	Prospective observational cohort Patient recruitment: April-May 2020 3-month follow-up: June–July 2020 8-month follow-up: December 2020 - January 2021 Vaccination: January - February 2021 Follow-up = 1-month post-vaccination	*n* = 66 Female = 25 Hospitalized = 66	Vaccinated: 64 (54–73) years Unvaccinated: 55 (47–60) years	44 vaccinated participants	22 un-vaccinated participants	Telephone interview of quality of life (SF-36), mental wellbeing (WEMWBS) and ongoing symptoms	Fatigue Breathlessness Insomnia ENT symptoms Brain fog Muscle aches Anosmia Joint pain Cough Headache Palpitations Chest pain Diarrhoea Abdominal pain Nausea	Product: BNT162b2, ChAdOx1 nCoV-19 Dose: One Follow-up: 1 month post-single vaccination	Worsening of symptoms Vaccinated: 9/159 (5.6%) Unvaccinated: 13/91 (14.3%) Unchanged symptoms Vaccinated: 113/59 (71.1%) Unvaccinated: 64/91 (70.3%) Improvement of symptoms Vaccinated: 37/159 (23.2%) Unvaccinated:14/91 (15.4%) *p* value = 0.035 Physical Composite Score - Median (IQR) Vaccinate*d* = 41 (27–50) Unvaccinated = 34 (28–48) *p* value = 0.3 Mental Composite Score Median (IQR) Vaccinate*d* = 48 (37–54) Unvaccinated = 38 (29–48) *p* value = 0.039 Warwick and Edinburgh Mental Wellbeing scores Median (IQR) Vaccinated 3 month = 51 (40–59) 6 month = 49 (42–57) Post-vaccination = 52 (41–61) Unvaccinated 3 month = 48 (38–54) 6 month = 45 (36–50) Matched post-vaccination = 54 (46–58)
Gaber et al. 2021^26^ United Kingdom	ND	*n* = 67 Females = ND Hospitalized = 67	18–65 years	67 healthcare workers with long-COVID-19	No control group	Survey questionnaire	Fatigue Shortness of breath Anxiety	Product: mRNA COVID-19 vaccine Dose: One dose Follow-up: At least 2 weeks post-single vaccination	Worsening of symptoms 8/67 (12%): 3 with fatigue, 1 with respiratory symptoms, 2 with anxiety, 2 with worsening of other symptoms No change in symptoms 45/67 (67%) Improvement of one or more symptoms 14/67 (21%): 8 improving respiratory symptoms, 4 improving fatigue, 5 improving anxiety, 2 improving other symptoms
Scherlinger et al. 2021^27^ United States of America	Cross sectional August 3-17, 2021	*n* = 567 Females = 473 Hospitalized = 25	44 (37-50) years	397 vaccinated with long-COVID-19 (255: 1 dose, 142: 2 doses) Hospitalized: 18	170 unvaccinated with long-COVID-19 Hospitalized: 7	Survey questionnaire	Fever/Chills Fatigue Brain fog Headaches Changing mood/Impact on morale Sleeping issues Costal pain Dyspnea Cough Palpitations Muscle aches Joint pain Paresthesia/Tingling Anosmia/Ageusia Diarrhoea/Vomiting Spontaneous bruises Pruritus	Product: BNT162b2, mRNA 1273, ChAdOx1 nCoV-19, Ad26.COV2.S, combination of mRNA/vector vaccine Dose: 1-2 Follow-up: Not reported	Improvement of symptoms after vaccination: 83 (21.8%) Anosmia 62% Brain fog 51% Worsening of symptoms after vaccination: 117 (31%) Fever/chills 74% GI symptoms 70% Paresthesia 64% Arthralgia 63%
Tsuchida et al. 2022^28^ Japan	Cohort Study period: ND	*n* = 42 Female = 25 Hospitalization = ND	45 (32–55) years	42 long COVID-19 patients	None	Self-assessments of post-vaccination changes in the main sequelae symptoms were confirmed based on the patient's response as follows: unchanged, relief, and worsened.	Fatigue Joint pain Taste and olfactory abnormality Numbness Sore throat Dizziness Memory impairment Palpitations Cough Headache Chest ache Anxiety	Product: Not reported Dose: One Follow-up: 2 weeks post-single vaccination	*n* (%) Fatigue Unchanged: 15(55.6) Relief: 5(18.5) Worse: 4(14.8) Joint pain Unchanged: 2(7.4) Worse: 2(7.4) Loss of Taste Unchanged: 5(18.5) Worse: 0(0)
Peghin et al. 2022^29^ Italy	Prospective cohort 6 months: September- November 2020 12 months: March–May 2021	*n* = 479 Overall Female: 252 (52.6) Vaccinated Female: 94 (71.2) Unvaccinated Female: 158 (45.5)	*n* (%) Overall: 18–40: 107 (22.3) 41–60: 205 (42.8) >60: 167 (34.9) Vaccinated: 18–40: 33 (25.0) 41–60: 64 (48.5) >60: 35 (26.5) Unvaccinated: 18–40: 74 (21.3) 41–60: 141 (40.6) >60: 132 (38.0)	132 vaccinated	347 unvaccinated	Telephone interviews	Fatigue Anosmia/dysgeusia Dyspnea Cough Chest pain Headache Rheumatological disorders Gastrointestinal disorders Cutaneous lesions Hair loss URTI symptoms Ocular symptoms Neurological disorders Psychiatric disorders	Product: BNT162b2, mRNA 1273, ChAdOx1 nCoV-19, Ad26.COV2.S Dose: At least one dose Follow-up: Not reported	Post-COVID symptoms at 12-months compared with 6-months by vaccination Post-COVID-19 syndrome (*p*=0.209) Vaccinated (*n*=132) Unchanged: 87 (65.9%) Worsened: 30 (22.7%) Improved: 15 (11.4%) Unvaccinated (*n*=347) Unchanged: 247 (71.2%) Worsened: 55 (15.8%) Improved: 45 (13.0%) Post-COVID symptoms, *n* (%) (*p*=0.604) Vaccinated (*n*=132) 0: 73 (55.3%) 1: 27 (20.4%) 2: 17 (12.9%) 3: 7 (5.3%) 4: 1 (0.8%) ≥5: 7 (5.3%) Unvaccinated: 0: 180 (51.9) 1: 65 (18.7) 2: 42 (12.1) 3: 27 (7.8) 4: 11 (3.2) >5: 22 (6.3)
Strain et al. 2022^30^ UK, Israel, Russia, India, South Africa	Cross- sectional March 16, 2021 and April 5, 2021	*n* = 812 Female = 80.6% Short hospital stay = 7.4% Long hospital stay +/- ITU = 3.6%	<20 to>71 years old	812 online Survey respondents	No control group	Survey questionnaire	Fatigue Brain Fog Myalgia Shortness of Breath Insomnia Chest Pain Gastrointestinal symptoms Anosmia Autonomic dysfunction Postural Orthostatic Tachycardia Syndrome Persistent Cough Fever Rash (incl. COVID-19 toes) Vascular complications	Product: ChAdOx1 nCoV-19, BNT162b2, mRNA 1273 Dose: One dose Follow-up: 1-21 weeks (median 9 weeks) post-single vaccination	57.9% reported overall improvement of symptoms 58% of participants vaccinated with ChAdOx1 nCoV-19 reported overall improvement of symptoms 56% of participants vaccinated with BNT162b2 reported overall improvement of symptoms 66% of participants vaccinated with mRNA 1273 reported overall improvement of symptoms 17.9% reported a worsening of their symptoms 19% of participants vaccinated with ChAdOx1 nCoV-19 reported worsening of their symptoms 18% of participants vaccinated with BNT162b2 reported deterioration of their average symptoms 12% of participants vaccinated with mRNA 1273 reported deterioration of their symptoms 24.2% reported no difference The mRNA 1273 vaccine compared favorably with ChAdOx1 nCoV-19 vaccine for improvements in fatigue (*p* = 0.009), brain fog (*p* = 0.01), myalgia (*p* = 0.006), gastro-intestinal symptoms (*p* = 0.05) and autonomic dysfunction (*p* = 0.004)
Ayoubkhani et al. 2022^31^ United Kingdom	Prospective cohort February 3 to September 5, 2021	*n* = 28,356 Female Overall =15,760, Standardized difference = -7.1 mRNA vaccine = 7393 Adenovirus vector vaccine = 8367 Hospital admission with COVID-19 = 900 Standardized difference = 4.0 mRNA vaccine = 359 Adenovirus vector vaccine = 541	18–69 years old Mean age: 46 years	Participants with long-COVID symptoms vaccinated with mRNA (*n*=12,859) Participants with long-COVID symptoms vaccinated with adenovirus vector (*n*=15,497)	ND	COVID-19 Infection Survey UK Government Statistical Office	Loss of smell Loss of taste Trouble sleeping Fatigue Headache Trouble sleeping	Product: ChAdOx1 nCoV-19, BNT162b2, mRNA 1273 Dose: 1 Dose, 2 Doses Follow-up: Median time from first vaccination 141 days (among all participants) Median time from second vaccination 67 days (83.8% of participants)	After dose 1 Loss of smell (OR −12.5%, −21.5% to −2.5%, *p*=0.02) Loss of taste (OR −9.2%, −19.8% to 2.7%, *p*=0.13) Trouble sleeping (OR −8.8%, −19.4% to 3.3%, *p*=0.15) After dose 2 Fatigue (OR −9.7%, −16.5% to −2.4%, *p*=0.01) Headache (OR −9.0%, −18.1% to 1.0%, *p*=0.08) Trouble sleeping (OR −9.0%, −18.2% to 1.2%, *p*=0.08).
Kuodi et al. 2022^32^ Israel	Cross- sectional March 2020 to November 2021	*n* = 3388 No. of participants who filled out ‘sex’: 750 Female Overall *n =* 467, *p=0.206* Received 1 dose = 175 Received 2 doses = 136 Unvaccinated = 156 Hospitalized Overall *n* = 85, *p* = 0.277 Received 1 dose = 35 Received 2 doses = 21 Unvaccinated = 29	≥ 18 years old	Received 1 vaccine dose (*n*=340) Received 2 vaccine doses (*n*=294)	Unvaccinated (*n=*317)	Survey Questionnaire - International Severe Acute Respiratory and emerging Infection Consortium (ISARIC)	Fatigue Headache Weakness in arms or legs Persistent muscle pain Loss of concentration Hair loss Sleeping problems Dizziness Persistent cough Shortness of breath	Product: BNT162b2 Dose: 1 dose group, 2 doses group Follow-up: Not reported	Fatigue (21.87%) Vaccinated, 1 dose (*n*=93) RR: 1.057 [0.820–1.364] Vaccinated, 2 doses (*n*=33) RR: 0.434 [0.299–0.629] *p-*value: 0.003 Unvaccinated (*n*=82) Headache (19.98%) Vaccinated, 1 dose (*n*=110) RR: 1.081 [0.814–1.435] Vaccinated, 2 doses (*n*=77) RR: 0.641 [0.450–.911] * Unvaccinated (*n*=95) Weakness arms/legs (13.5%) Vaccinated, 1 dose (*n*=127) RR: 1.042 [0.738–1.472] Vaccinated, 2 doses (*n*=82) RR: 0.423 [0.258–0.692] * Unvaccinated (*n*=103) Muscle pain (10.3%) Vaccinated, 1 dose (*n*=106) RR: 1.165 [0.773–1.757] Vaccinated, 2 doses (*n*=80) RR: 0.509 [0.292–0.886] * Unvaccinated (*n*=86) Loss of concentration (9.5%)
									Vaccinated, 1 dose (*n*=59) RR: 1.243 [0.893–1.901] Vaccinated, 2 doses (*n*=48) RR: 0.425 [0.228–0.791] * Unvaccinated (*n*=55) Hair loss (9.25%) Vaccinated, 1 dose (*n*=43) RR: 1.113 [0.735–1.687] Vaccinated, 2 doses (*n*=9) RR: 0.270 [0.132–0.550] * Unvaccinated (*n*=36) Sleeping problems (8.94%) Vaccinated, 1 dose (*n*=42) RR: 1.350 [0.863–2.113] Vaccinated, 2 doses (*n*=14) RR: 0.521 [0.281–0.965] * Unvaccinated (*n*=29) Dizziness (7.78%) Vaccinated, 1 dose (*n*=30) RR: 0.874 [0.544–1.404] Vaccinated, 2 doses (*n*: 12) RR: 0.404 [0.212–0.770] * Unvaccinated (*n*=32) Persistent cough (7.36%) Vaccinated, 1 dose (*n*=26) RR: 1.010 [0.593–1.711] Vaccinated, 2 doses (*n*=20) RR: 0.899 [0.507–1.592] Unvaccinated (*n*=26) Shortness of breath (7.15%) Vaccinated, 1 dose (*n*=29) RR: 1.081 [0.648–1.805] Vaccinated, 2 doses (*n*=14) RR: 0.604 [0.320–1.139] Unvaccinated (*n*=25)
Nehme et al. 2022^33^ Switzerland	Prospective cohort April 23 to July 27, 2021	*n = 1596* Female = 883 Males= 713 all participants are out-patient	Mean age: 43.5 years	771 vaccinated (424 first dose, 347 second dose)	825 unvaccinated	REDCap v11.0.3 and Stata 15.1 (StataCorp)	Fatigue Difficulty concentrating or memory loss Loss or change in smell Loss or change in taste Shortness of breath Headache	Product: BNT162b2, mRNA 1273 Dose: 1-2	Vaccination (one or two doses) was associated with decreased prevalence of the six cardinal post-COVID symptoms [aPR 0.72; 0.56–0.92] Vaccination with 2 doses decreased prevalence of dyspnea [aOR 0.34; 0.14–0.82]and change in taste [aOR 0.38; 0.18-0.83] Decreased prevalence of any one symptom [aOR 0.60; 0.43–0.83]
Tran et al. 2021^34^ France	Prospective cohort November 2020 to May 2021 (still ongoing)	*n* = 910 Female = 733 Male = 177 Hospitalized = 81	Mean age: 47 years	445 vaccinated	455 unvaccinated	ComPaRelong-COVID-19 database	COVID-19 ST score (53 symptoms)	Product: BNT162b2, mRNA 1273, ChAdOx1 nCoV-19 Dose: 1–2	Long-COVID was significantly less severe in the vaccination group than in the control group mean (SD) long-COVID ST score 13 (9.4) in the vaccination group and 14.8 (9.8) in the control group Mean Difference: -1.8, 95% CI -2.5 to -1.0 16.6% complete remission from long-COVID 7.5% (control group)
Wisnivesky et al. 2022^35^ United States of America	Prospective Cohort Patient recruitment: July 20, 2020 - February 26, 2021 6-month interview: August 23, 2021	*n* = 453 Female *n* = 294 Hospitalizedpatients (ER, Inpatient, ICU) *n* = 264	mean (SD) Vaccinated = 50.1 (13.4) years Unvaccinated = 49.7 (14.1) years	324 vaccinated participants	129 unvaccinated participants	5-point Likert question for anosmia Modified Medical Research Council (mMRC) scale for dyspnea St. George's questionnaire for respiratory symptoms Patient Health Questionnaire-8 (PHQ-8) for depression Generalized Anxiety Disorders-7 (GAD-7) instrument for anxiety PTSD checklist for DSM-5 (PCL-5) for PTSD symptoms Patient-Reported Outcomes Measurement Information System (PROMIS)-29 v2.0 Scale for quality of life	Anosmia Respiratory symptoms Dyspnea Cough Phlegm Wheezing Depression symptoms Anxiety symptoms COVID-19 PTSD symptoms Non-COVIS-19 PTSD symptoms Quality of life Physical function Anxiety Depression Fatigue Social roles Sleep Pain	Product: BNT162b2, mRNA 1273, Ad26.COV2.S Dose: at least one dose of vaccine Follow-up: 2 weeks - 6 months post single vaccination	Difference change vaccinated *vs.* unvaccinated (95% CI) Anosmia -0.26 (-0.54 to -0.03) Respiratory symptoms Dyspnea 0.02 (-0.19 to 0.23) Cough 0.003 (−0.39 to −0.39) Phlegm -0.28 (−0.76 to 0.20) Wheezing 0.41 (−0.27 to 1.1) Depression symptoms 0.32 (−0.88 to −1.53) Anxiety symptoms 1.29 (−0.24 to −2.82) COVID-19 PTSD 3.41 (−1.82 to −8.63) Quality of life Physical function −0.95 (−2.96 to 1.05) Fatigue -1.40 (−3.98 to 1.18) Social role -2.32 (−5.51 to −0.87) Sleep 1.16 (−1.10 to - 3.41) Pain −0.84 (−3.19 to 1.52)

ND - no data; aOR - adjusted odds ratio; SD - standard deviation; OR - odds ratio; HR - hazard ratio; RR - risk ratio; BTI - breakthrough infections; ICU -intensive care unit; PTSD - post-traumatic stress disorder; ER - emergency room.

There was heterogeneity in the presentation of results for the ‘infection-long COVID-vaccine’ studies. Six out of the 11 articles^25^, ^26^, ^27^, ^28^, ^29^, ^30^ made use of percentage in reporting the outcomes, one study^31^ used OR, one^33^ aOR, one^35^ mean difference, one^32^ risk ratio (RR), and the last one^34^ all measures: mean difference, HR, and risk difference for the presentation of results. Seven articles^26^^,^^27^^,^^30^, ^31^, ^32^, ^33^, ^34^ agreed that there was improvement in long-COVID symptoms at least one dose post-vaccination, two of which^30^^,^^32^ reported that two doses of vaccines restored the reported symptoms back to baseline. On the contrary, four studies^25^^,^^28^^,^^29^^,^^35^ reported no change of long-COVID symptoms in the majority of participants. Tran et al.^34^ stated that vaccination doubled the remission rate of long-COVID. On the contrary, Tsuchida et al.^28^ noted that those participants worsening their long-COVID symptoms were reported to have increased antibody titer ratio resulting from excessive immune response to vaccination.

Seven out of the 11 articles^28^, ^29^, ^30^, ^31^, ^32^, ^33^^,^^35^ listed changes in post-acute symptoms manifested by the patients, while 5 studies^25^, ^26^, ^27^^,^^30^^,^^33^ reported improvement, unchange, or worsening of the long-COVID symptoms. The most common long-COVID symptoms evaluated in the ‘infection-long COVID-vaccine’ papers were fatigue (*n*=6), anosmia (*n*=6), and dysgeusia (*n*=4). Neurological symptoms and mental health problems including headache (*n*=5), anxiety (*n*=4), depression (*n*=2), brain fog (*n*=2), insomnia (*n*=2) and memory loss (*n*=1) were also reported.

Finally, the definition of long-COVID was not consistent. Seven articles described long-COVID in accordance with the WHO^4^ as having COVID-19 symptoms usually 3 months from the onset of COVID-19 and that lasts for at least 2 months and cannot be explained by an alternative diagnosis.^19^^,^^22^^,^^28^, ^29^, ^30^, ^31^, ^32^ Two papers defined long-COVID in having persistent symptoms lasting for more than 4 weeks and the lack of an alternative diagnosis,^20^^,^^27^ and the remaining articles did not specify a particular definition of long-COVID, doing follow-up periods ranging from 1 month to 6 months after hospital discharge.^21^^,^^23^, ^24^, ^25^, ^26^^,^^33^, ^34^, ^35^, ^36^, ^37^, ^38^

### Methodological quality

Two studies (11.8%)^20^^,^^27^ used a case-control design and were of high (8/9 stars) and medium methodological quality (6/9 stars). The remaining fifteen (88.2%) were cohort studies, with six using a cross-sectional^21^^,^^26^^,^^28^^,^^30^^,^^32^^,^^33^ (*n*=6/17, 35.3%) and nine a longitudinal^19^^,^^22^^,^^24^^,^^25^^,^^29^^,^^31^^,^^34^^,^^35^^,^^38^ (*n*=9/17, 52.9%) design. Fourteen were of high methodological quality (3/3 stars or 7/9 stars, as appropriate) and one was of medium quality (6/9 stars). No disagreement between authors was observed. Table 3, Table 4 present the Newcastle-Ottawa Scale scores for each study and a summary of every item.

**Table 3:** Newcastle - Ottawa quality assessment scale evaluating methodological quality/risk of bias (case-control studies).

	Selection				Comparability		Exposure			
Study	Adequate case definition	Representativeness of cases	Selection of controls	Definition of controls	Controlled for age	Controlled for additional factors	Ascertainment of exposure	Same method for cases and controls	Non-response rate	Score
Scherlinger et al. 2022^27^		★	★		★	★	★	★		6/9
Antonelli et al. 2022^20^	★	★	★	★	★	★	★	★		8/9

**Table 4:** Newcastle - Ottawa quality assessment scale evaluating methodological quality/risk of bias (cross-sectional or longitudinal descriptive studies and cohort studies).

	Selection		Comparability				Exposure			
Study	Representativeness of the exposed cohort	Selection of the non-exposed cohort	Ascertainment of exposure	Outcome of interest	Controlled for age	Controlled for additional factors	Assessment of outcome	Follow-up long enough	Adequacy of follow-up	Score
Gaber et al. 2020^26^	★		★				★			3/3
Senjam et al. 2021^21^	★		★				★			3/3
Nehme et al. 2021^33^	★		★				★			3/3
Kuodi et al. 2022^32^	★		★				★			3/3
Tsuchida et al. 2021^28^	★		★				★			3/3
Strain et al. 2022^30^	★		★				★			3/3
Peghin et al. 2022^29^	★	★	★	★			★	★	★	7/9
Tran et al. 2022^34^	★	★		★		★	★	★	★	7/9
Ayoubkhani et al. 2022^31^	★	★	★	★			★	★	★	7/9
Ayoubkhani et al. 2022^22^	★	★		★	★	★	★	★	★	8/9
Wisnivesky et al. 2022^35^	★	★	★	★			★	★	★	7/9
Simon et al. 2021^19^	★	★		★			★	★	★	6/9
Taquet et al. 2021^24^	★	★		★		★	★	★	★	7/9
Al-Aly et al. 2022^23^	★	★	★	★			★	★	★	7/9
Arnold et al. 2020^25^	★	★	★	★		★	★	★	★	8/9

## Discussion

This is the first systematic review to date aimed at summarising data about the impact of COVID-19 vaccine on long-COVID, to our knowledge. Low level of evidence (grade III, case-controls, cohort studies) suggests that vaccination before SARS-CoV-2 infection could reduce the risk of subsequent long-COVID; however, the influence of vaccination in people with previous long-COVID remains controversial, with evidence reflecting symptoms improving and others not. Our results agree with current opinions questioning the real impact the vaccines may have on current long-COVID symtptoms.^13^, ^14^, ^15^, ^16^^,^^40^

The first situation is to assess if vaccines prevent long-COVID development. We identified six level III studies of moderate to high methodological quality investigating if vaccination before SARS-CoV-2 acute infection reduces the risk of developing long-COVID after (vaccine-infection-long COVID design). All studies found that vaccines reduced the risk of developing long-COVID in people with mild to moderate COVID-19, supporting the hypothesis that vaccination could be used as a preventive strategy for reducing long-term symptoms. However, most studies assessed the “short-term” effect of vaccines, since most included patients infected from one week to one month after vaccination. Only two studies investigated follow-up periods of six months after vaccination.^23^^,^^24^ Further, the definition of long-COVID was inconsistent between studies. Additionally, preliminary data suggest that two doses could be more effective than one single dose^24^ and that BNT162b2 (“Pfizer/BioNTech”) or mRNA-1273 (“Moderna”) vaccine could be more effective than Ad26.COV2.S (“Janssen”) vaccine^24^ for reducing the risk of developing long-COVID, in keeping with previous data showing that the efficacy of mRNA-based vaccines on the risk of developing severe illness may be higher compared to adenoviral vaccines. No study investigated the impact of vaccine boosters on long-COVID.

The mechanisms underlying a potential risk reduction of long-COVID in people previously vaccinated are unknown. Two hypotheses are proposed. First, since vaccines reduce the severity of acute SARS-CoV-2 infection, this may then translate into lower risk of developing organ or systemic derangements, and thus symptoms onset and duration. However, the association of long -COVID with COVID-19 severity remains controversial.^41^ A second hypothesis is that vaccines may accelerate clearance of the remaining SARS-CoV-2 virus in the human body (viral remnant hypothesis of long-COVID) or could also reduce the exaggerated inflammatory and/or immune response associated with long-COVID development (immune/inflammatory hypothesis of long-COVID).^42^ Future studies investigating the underlying mechanisms of vaccines on long-COVID would be needed to clarify these issues.

The second topic is to know if COVID-19 vaccines represent a risk for those individuals with ongoing long-COVID symptomatology. We identified eleven level III studies of moderate to high methodological quality investigating the impact of vaccine on individuals who had previously suffered from COVID-19 and developed long-COVID (infection-long COVID-vaccine design). The results here were less consistent, since 63% of the studies (*n*=7/11) found that vaccination improved ongoing symptoms of long-COVID, whereas 36% (*n*=4/11) reported small changes or even worsening in some patients. Again, the definition of long-COVID among the studies was inconsistent. This heterogeneity in the response against vaccines of individuals with long-COVID could be related to the complexity of this condition. For instance, Tsuchida et al.^24^ identified that people experiencing a worsening of long-COVID symptoms after vaccination are those also showing excessive immune response to vaccination, with higher increased rate of antibody titers. On the contrary, Peghin et al.^24^ observed that COVID-19 vaccines did not produce an altered humoral response in individuals with current long-COVID. Discrepancies between these studies could be related to the fact that numerous autoantibodies may be produced after SARS-CoV-2 infection^43^ and, accordingly, COVID-19 vaccines effects could be dependent on the host immune response. Further, since long-COVID includes a myriad of >100 different multiorgan symptoms,^5^ it is possible that vaccines influence could be related to some specific long-COVID symptoms. Accordingly, COVID-19 vaccination may help to reduce long-COVID by eradicating the viral reservoir or by resetting a deregulated immune response to primary acute infection, and this effect could be host-dependent. Overall, although current evidence is inconclusive, available data suggest that COVID-19 vaccines are important factors for further immunological protection against potential reinfections.

The results of this systematic review should be considered according to potential strengths and limitations. Among the strengths, we conducted a deep systematic search of all the available evidence about the impact of vaccines on long-COVID. This led to identification of six non-peer reviewed, preprint articles. Considering the rapid emergence which represents the COVID-19 pandemic, the volume of preprint research could be expected given the need for rapid data dissemination. Second, this is the first time that the methodological quality of published studies is conducted. Interestingly, albeit heterogeneity in the concepts and designs, the quality of most study designs (82%) was high.

Three main limitations should be recognised. First, the effects of vaccines on long-term post-COVID symptoms are scarce, since most studies identified in this review investigated the risk of long-COVID in people infected the first month after being vaccinated. Second, there was no consistent definition of long-COVID in the published literature. In most studies, symptoms were assessed during the first month after the infection, which could not represent the reality of long-COVID, where symptoms can persist during months and years.^9^^,^^10^ We included all studies investigating changes in any symptom appearing after a SARS-CoV-2 infection. In fact, just seven studies (41%) used the WHO definition of post-COVID-19 condition.^4^ Future studies including the WHO definition of post-COVID-19 condition^4^ should be conducted to get better stratification of the population. In addition, it should be considered that vaccinated individuals were older than non-vaccinated, probably because worldwide vaccination strategies firstly focused on vulnerable individuals. Third, no study differentiated between hospitalised and non-hospitalised patients or sex-differences between males and females. Similarly, no evidence is available on the SARS-CoV-2 variants that caused acute infections, since no study summarise the VoC included in their population samples; so that a bias on long-COVID burden and characteristics attributable to infection with different VOCs cannot be ruled out. Therefore, studies investigating the impact of COVID-19 vaccines in 1, hospitalised or non-hospitalised patients; 2, males and females; and 3, the different VoC and potential reinfections are now needed. Finally, no study investigated the impact of vaccine boosters in long-COVID symptomatology. Since booster programs have been increasingly implemented in several countries, particularly in vulnerable individuals, the impact of third or fourth booster dose on long-COVID should be investigated.

In conclusion, low level of evidence suggests that vaccination before SARS-CoV-2 infection could reduce the risk of developing subsequent long-COVID. It seems that two doses of vaccine could be more effective than just one dose, although data are preliminary and based in just two studies. No data on vaccine boosters are still available. The impact of vaccination in people who had been infected, had developed long-COVID symptoms, and, then vaccinated is inconsistent, with both positive and negative impact. This conclusion is based on grade III studies (case-controls, cohort studies). These assumptions are also limited to those vaccines used in the studies. This highlights the need for more studies better defining the participants involved, the inclusion of different SARs-CoV-2 VoC, and a proper definition of long-COVID.

## Contributors

All the authors cited in the manuscript had substantial contributions to the concept and design, the execution of the work, or the analysis and interpretation of data; drafting or revising the manuscript and have read and approved the final version of the paper. Kin Israel Notarte: conceptualisation, visualisation, methodology, validation, formal analysis, data curation, writing-original draft, writing-review and editing, conceptualisation, formal analysis, data curation, writing-review and editing. Jesus Alfonso Catahay: methodology, validation, formal analysis, data curation, writing-original draft, writing-review and editing. Jacqueline Veronica Velasco: methodology, validation, formal analysis, data curation, writing-original draft, writing-review and editing. Adriel Pastrana: methodology, validation, formal analysis, data curation, writing-original draft, writing-review and editing. Abbygail Therese Ver: methodology, validation, formal analysis, data curation, writing-original draft, writing-review and editing. Flos Carmeli Pangilinan: methodology, validation, formal analysis, data curation, writing-original draft, writing-review and editing. Princess Juneire Peligro: methodology, validation, formal analysis, data curation, writing-original draft, writing-review and editing. Michael Casimiro: methodology, validation, formal analysis, data curation, writing-original draft, writing-review and editing. Jonathan Jaime Guerrero: writing-review and editing. Ma. Margarita Leticia Gellaco: writing-review and editing. Giuseppe Lippi: writing—review and editing. Brandon Michael Henry: writing-review and editing César Fernández-de-las-Peñas: conceptualisation, visualisation, validation, formal analysis, writing-review and editing, and supervision. All authors had access to the data. Kin Israel Notarte and César Fernández-de-las-Peñas verified the data set. All authors were responsible for making the decision to submit this manuscript.

## Data Sharing Statement

All data derived from this study are in the article.

## Declaration of interests

The authors declare no conflict of interest.

## Appendix Supplementary materials

Image, application 1
