# Supplementary material for: Impact of COVID-19 vaccination on the risk of developing long-COVID and on existing long-COVID symptoms: A systematic review
Source: eClinicalMedicine. 2022 Aug 27;53:101624. doi: 10.1016/j.eclinm.2022.101624 (PMC9417563; doi:10.1016/j.eclinm.2022.101624)
Supplement: Supplementary file 1 [file mmc1.pdf]

Supplementary Table: Database formulas during literature search

**PubMed Search Formula**
#1 "post-acute COVID-19 syndrome" [MeSH Terms] OR "long-COVID" [All Fields] OR "long-COVID symptoms" [All Fields] OR "long hauler" [All Fields] OR "post-COVID-19" [All Fields] OR "post-acute COVID-19 symptoms" [All Fields] OR "COVID-19 sequelae" [All Fields]<br>#2 "COVID-19 vaccines" [MeSH Terms] OR "vaccines"[MeSH Terms] OR "vaccination" [MeSH Terms] OR "SARS-CoV-2 vaccines" [All Fields]<br>#3 #1 AND #2
**Medline / CINAHL (via EBSCO) Search Formula**
#1 "post-acute COVID-19 syndrome" OR "long-COVID" OR "long-COVID symptoms" OR "long hauler" OR "post-COVID-19" OR "post-acute COVID-19 symptoms" OR "COVID-19 sequelae"<br>#2 "COVID-19 vaccines" OR "vaccines" OR "vaccination" OR "SARS-CoV-2 vaccines"<br>#3 #1 AND #2
**WOS (EMBASE) / Web of Science Search Formula**
("post-acute COVID-19 syndrome" OR "long-COVID" OR "long-COVID symptoms" OR "long hauler" OR "post-COVID-19" OR "post-acute COVID-19 symptoms" OR "COVID-19 sequelae" AND ("COVID-19 vaccines" OR "vaccines" OR "vaccination" OR "SARS-CoV-2 vaccines")
